# Early Training-Induced Reduction of Angiotensinogen in Autonomic Areas—The Main Effect of Exercise on Brain Renin-Angiotensin System in Hypertensive Rats

**DOI:** 10.1371/journal.pone.0137395

**Published:** 2015-09-15

**Authors:** Laiali Jurdi Chaar, Tatiana Pereira Alves, Alvaro Martins Batista Junior, Lisete Compagno Michelini

**Affiliations:** 1 Department of Physiology and Biophysics, Institute of Biomedical Sciences, University of Sao Paulo, Sao Paulo, SP, Brazil; 2 Department of Anatomy, Institute of Biomedical Sciences, University of Sao Paulo, Sao Paulo, SP, Brazil; University of São Paulo, BRAZIL

## Abstract

**Background:**

Exercise training (T) blunts functional deficits and renin-angiotensin system (RAS) hyperactivity in hypertensive individuals. There is no information on T-induced temporal changes of brain RAS. We evaluate now the simultaneous effects of T on functional responses and time course changes in the expression/activity of brain RAS components in autonomic cardiovascular-controlling areas.

**Methods and Results:**

Spontaneously hypertensive rats (SHR) and age-matched normotensive controls (WKY) were trained for 0, 1, 2, 4, 8 and 12 weeks. Sedentary (S) groups served as time-controls. After arterial pressure (AP) and heart rate (HR) recordings at rest, fresh and fixed brains were harvested for qPCR and immunofluorescence assays. SHR-S *vs*. WKY-S exhibited higher mean AP (MAP) and HR, increased pressure variability and sympathetic activity, elevated AT_1_ receptor (AT_1_) expression in nucleus tractus solitarii (NTS) and higher Mas receptor expression in the rostroventrolateral medulla (RVLM). In SHR, T promptly (T_2_ on) reduced sympathetic variability to heart/vessels and largely decreased angiotensinogen expression in the paraventricular hypothalamic nucleus (PVN) and NTS, with a late RVLM reduction (T_4_). AT_1_ expression was only reduced at T_12_ (PVN and NTS) with transient, not maintained Mas receptor changes in PVN and RVLM. These responses were accompanied by baseline MAP and HR reduction in the SHR-T (from T_4_ on). In the SHR group, PVN angiotensinogen expression correlated positively with sympathetic activity, resting MAP and HR. In WKY-T, a precocious (T_2_-T_12_) RVLM AT_1_ decrease preceded the appearance of resting bradycardia (from T_8_ on).

**Conclusions:**

Early and maintained reduction of angiotensinogen content in autonomic areas of the SHR is the most prominent effect of training on brain RAS. Down-regulation of PVN RAS expression is an essential factor to drive cardiovascular benefits in SHR-T, while resting bradycardia in WKY-T is correlated to RVLM AT_1_ reduction.

## Introduction

Chronic hypertension is closely related to overactivity of the pressor axis of the renin-angiotensin system (RAS) [1–3]. Angiotensin II (Ang II), through its vasoconstrictor, trophic, inflammatory and anti-natriuretic effects, increases total peripheral resistance and blood volume, being a major contributor for the establishment/maintenance of hypertension[4]. Previous reports showing high levels of precursor, enzymes, peptides and/or receptors in cardiovascular controlling areas of hypertensive individuals suggested that brain RAS is involved in the pathogenesis of hypertension [2, 4, 5]. Indeed, the increased expression of angiotensinogen (Aogen), angiotensin converting enzyme (ACE) and angiotensin type 1 receptor (AT_1_) and the elevated Ang II content in autonomic brain areas, reduce baroreceptor reflex control, alter the sympatho-vagal balance to the heart and cause a robust increase in sympathetic nerve activity[4, 6, 7]. These observations confirmed the involvement of brain ACE-Ang II-AT_1_ axis in the pathophysiology of hypertension.

Over the last decade, ACE2 has emerged as a new player in the RAS: it metabolizes Ang II into angiotensin-(1–7) [Ang-(1–7)] that acts on Mas receptor determining effects different from and often opposing those of the AT_1_ [8–11]. The ACE2-Ang-(1–7)-Mas receptor axis mediates vasodilatory, anti-trophic, anti-inflammatory and natriuretic actions, thus counterbalancing the effects of the pressor axis. In recent years, it became apparent that the balance between the opposing effects mediated by Ang II and Ang-(1–7), may have a pivotal role in determining different cardiovascular pathophysiologies[9]. Accumulated experimental evidence has shown that both axes contribute to the autonomic control of the circulation and that hypertensive and elder individuals exhibited a clear unbalance, with the predominance of Ang II-mediated over Ang-(1–7)-mediated effects[5, 12].

In our hands, Ang II overactivity in chronic hypertension was associated with decreased sensitivity of baroreceptors’ afferents, altered nucleus tractus solitarii (NTS) integration; increased sympathetic outflow to heart and vessels, reduced reflex control of the heart and increased baseline heart rate[13–17]. These effects were associated with augmented Aogen and AT_1_ expression, higher oxidative stress and increased pro-inflammatory cytokines expression in autonomic brain areas as the NTS, the rostroventrolateral medulla (RVLM) and the paraventricular nucleus of the hypothalamus (PVN)[5, 18–20]. Importantly, Aogen, ACE and AT_1_ expression as well as all Ang II-mediated responses were similarly blocked by both chronic treatment with AT_1_ antagonists and exercise training[5, 18–20]. Training also augmented ACE2 and Mas receptor expression within the RVLM and PVN of hypertensive rats[20]. Indeed exercise training is an efficient and safe tool to counteract deleterious effects induced by hypertension and other cardiovascular diseases[21]. Interestingly, recent studies by us indicated that training-induced effects on cardiovascular responses exhibit a sequential profile and differ between tissues. For example it was shown that trained-induced down-regulation of vascular RAS occurred in the renal but not in the femoral artery[22] and that normalization of oxidative and pro-inflammatory profile within the PVN of trained hypertensive rats was a fast response, preceding the changes in autonomic control of the heart[19]. There is no information on time-course changes induced by training on RAS expression/activity in autonomic brain areas. Knowing that NTS and RVLM act in concert with PVN to determine autonomic control of heart and vessels and that these areas are modulated by brain RAS, we hypothesize that training would promptly modify the expression of RAS precursor and receptors in autonomic brain areas and that these changes could contribute to training-induced responses on cardiovascular control. Therefore, we quantify, in trained (T) spontaneously hypertensive rats (SHR) and age-matched normotensive controls (WKY), time course changes in blood pressure and heart rate, sympathetic and parasympathetic activity to heart and sympathetic activity to vessels, simultaneously with changes in the expression of Aogen, AT_1_ and Mas receptor within the PVN, NTS and RVLM. Sedentary (S) SHR and WKY were used as time-control.

## Material and Methods

All surgical procedures and experimental protocols complied with the Ethical Principles in Animal Research of the Brazilian College of Animal Experimentation and were reviewed and approved by the Institutional Animal Care and Use Committee of the University of Sao Paulo.

### Animals and training protocols

Male 3-month old SHR and WKY rats were housed in the Animal Facilities of the Department of Physiology & Biophysics, Biomedical Sciences Institute at a controlled room temperature, with a 12:12 h light:dark cycle and free access to tap water and food. Rats were preselected for their ability to walk/run in a treadmill and were allocated to aerobic training (T, 50–60% of maximal exercise capacity, 5 days/week, 1 h/day[22]) or kept sedentary (S). Rats that did not run or stopped running during T protocol were excluded from the analysis.

### Arterial pressure, heart rate and autonomic measurements at rest

At the established time points (weeks 0, 1, 2, 4, 8 and 12 for T-groups and weeks 0 and 12 for S-groups), rats (12-18/sub-group) were anesthetized with ketamine plus xylazine (100 mg.kg^-1^ + 20 mg.kg^-1^, *ip*.*)* for chronic catheterization of the femoral artery. Direct continuous recordings of baseline arterial pressure (AP) and heart rate (HR) were made in conscious rats resting in their home cages at least 30-hours after the last training session and 24-hours after the catheterization[22]. Pulsatile arterial pressure (AP) was acquired on a beat-to-beat basis for ~40 min (2 kHz sampling frequency, IBM/PC computer, Lab Chart Pro, AD Instruments, Bella Vista, Australia) after the stabilization of cardiovascular parameters. HR was determined from pulse interval between two systolic peaks.

Time series of resting systolic AP (SAP) and pulse interval (PI) were used to calculate pressure and HR variability and their spectral components on the frequency domain: *very low frequency* (VLF < 0.02 Hz, indicative of hormonal and circadian changes), *low frequency* (LF = 0.2–0.75 Hz, indicating mainly the sympathetic activity to vessels and heart) and *high frequency* (HF = 0.75–3.0 Hz, indicative of vagal activity to the heart). The sympatho-vagal balance to the heart was assessed by LF/HF ratio of PI variability[23]. Power spectral analysis was performed on 10 min series of SAP and PI, using a customized software (*CardioSeries* from http://sites.google.com/site/cardioseries) as previously described[24].

### Tissue sampling

At weeks 0, 1, 2, 4, 8 and 12, after the functional measurements, rats (8-10/subgroup) were euthanized (overdose of ketamine/xylazine) and perfused transcardiacally with phosphate-buffered saline (PBS) immediately after the respiratory arrest[25]. Brain removal and bilateral “punching” of the PVN, NTS and RVLM were made as previously described[25]. Samples were stored in TRIzol at -80°C. For immunofluorescence assay, brains (3-4/subgroup) were initially perfused with PBS (5 min) followed by 4% paraformaldehyde (PFA, 20 ml/min, 400–500 ml), post-fixed in PFA for 4 h, cryoprotected (20% sucrose in 0.01 M PBS, 4°C for 3–4 days) and stored at -80°C until processing.

### Quantitative PCR (qPCR)

mRNA expression was measured in 7–10 samples/group, as described previously[25]. Briefly, total RNA was extracted and quantified by spectrophotometry. RNA (integrity confirmed by agarose gel electrophoresis) samples (2 μg) treated with DNase I and pure RNA were submitted to the reverse transcriptase reaction with RNaseOUT protection. Samples (in duplicates) were submitted to qPCR amplification using Platinum SYBR qPCR Supermix-UDG and specific oligonucleotides for Aogen (forward, 5’-CAC GGACAGCACCCTATTTT-3’; reverse 5’-TGTTGTCCACCCAGAACTCA-3’; 101 bp), AT_1_ (forward 5’-CCTCTACGCCAGTGTGTTCC-3’; reverse, 5’-GCC AAGCCAGCCATCAGC-3’; 114 bp) or Mas receptor (forward 5´- GGTGGAGAAAAGCAAGGACA-3'; reverse 5'—ACTGTCGGGCGGTCATCATC—3'; 263 bp). Hypoxanthine-guanine fosforibosil transferase (HPRT, forward, 5’-TTTGCTGACCTGCTGGATTAC-3’; reverse, 5’-ACTTTTATGTCCCCCGTTGA-3’; 124 bp), continually expressed in all samples and not changed by hypertension or training[19], was used as the reporter gene. Primers, designed with Primer-BLAST program (PubMed website), were purchased from Life Technologies. Standard curves for all genes were obtained with serial dilutions to determine the reaction’s efficiency. The mRNA expression in PVN, NTS and RVLM was calculated by cycle threshold (Ct) values using the ΔΔCt method[26] and the results were expressed as fold change from the WKY-S_0_ reference group, as described previously[26].

### Immunofluorescence

To confirm PCR data, fixed brains of some rats were processed for Aogen, AT_1_ and Mas receptor immunoreactivity. Sequential coronal sections of PVN, NTS and RVLM (30 μm, cryostat Leica, Model SM2000, Wetzlar, Germany) stored in antifreeze medium at -20°C, were processed. Briefly, free-floating sections, pretreated with 1% sodium borohydride and 1% H_2_O_2_ containing 10% of methanol, were incubated with 2% normal donkey serum (30 min) and then with primary antibodies diluted in PBS containing 2% normal donkey serum and 0.3% Triton X-100 (48 h at 4°C). The primary antibodies were monoclonal anti-rabbit Aogen (Epitomics, Burlingame, USA, 1:250), polyclonal anti-goat AT_1_ (Santa Cruz, Santa Cruz, USA, 1:50) or polyclonal anti-rabbit Mas receptor (Sigma Aldrich, USA, 1:1000), which were incubated together with monoclonal anti-mice NeuN (Millipore Corporation, Billerica, USA, 1:750) for 48 hours. Slices were washed and incubated (60 min, room temperature) with secondary antibodies: fluorescein-conjugated-FITC (Jackson ImmunoResearch, 1:100 donkey anti-rabbit or donkey anti-goat) and rhodamine-conjugated (Alexa 594, Molecular Probes, donkey anti-mouse 1:250). Negative controls omitted one or both primary antibodies or the secondary antibody. Sections were mounted on gelatin-coated slides plus coverslip and Vectashield (Vector Laboratories, Burlingame, USA) in a dark room and analyzed by a blind investigator. The images were captured using an optical microscope (200x magnification, Leica DFC 300 FX, Wetzlar, Germany).

### Statistical Analysis

Results are reported as means ± SEM. Training effects were analyzed by multivariate analysis (Multi-factorial ANOVA, STATISTICA 7 software, StatSoft, Inc, São Caetano do Sul, Brazil) comparing two factors: strain (WKY *vs*. SHR) and time (weeks 0, 1, 2, 4, 8 and 12). The comparison between groups (WKY *vs*. SHR) and conditions (sedentary *vs*. trained) at different time-points (week 0 *vs*. week 12) was made by three-way ANOVA. Fisher’s was used as the *post-hoc* test. Correlation analyses were performed using Pearson statistics. Differences were considered significant at *P*<0.05.

## Results

### Time course changes on treadmill performance

SHR exhibited a better treadmill performance than WKY since the beginning of protocols (Table 1). In both trained groups, significant and parallel increases on performance were observed at the 6^th^ week, with further increase from 6^th^ to 12^th^ week of training. At the end of protocols, performance gain was similar in SHR-T and WKY-T. On the other hand, 12 weeks of inactivity caused a small but significant decrease on treadmill performance in both sedentary groups (Table 1).

**Table 1:** Time-course change on treadmill performance (km.h^-1^) induced by sedentary (S) and training (T) protocols in WKY and SHR groups.

	WKY-S	WKY-T	SHR-S	SHR-T
**week 0**	0.98±0.06 (20)	1.02±0.03 (66)	1.62±0.08 (22) *	1.62±0.04 (74) *
**week 6**	0.72±0.14 (10) ●	1.32±0.06 (20) ●†	1.09±0.19 (11) *●	2.09±0.09 (27) *●†
**week 12**	0.77±0.07 (9) ●	1.92±0.12 (13) ●†	1.01±0.13 (15) ●	2.40±0.11 (12) *●†
**Gain**	-0.30±0.10 (9) ●	+0.88±0.14 (13) ●†	-0.43±0.16 (15) ●	+0.78±0.22 (12) ●†

Values are means±SEM. In parenthesis are the number of rats. Factorial ANOVA with repeated measurements. Significances (P<0.05): * (*vs* WKY), † (*vs* S); ● (*vs* week 0).

### Time course changes on baseline arterial pressure and heart rate and autonomic balance

**Fig 1:**
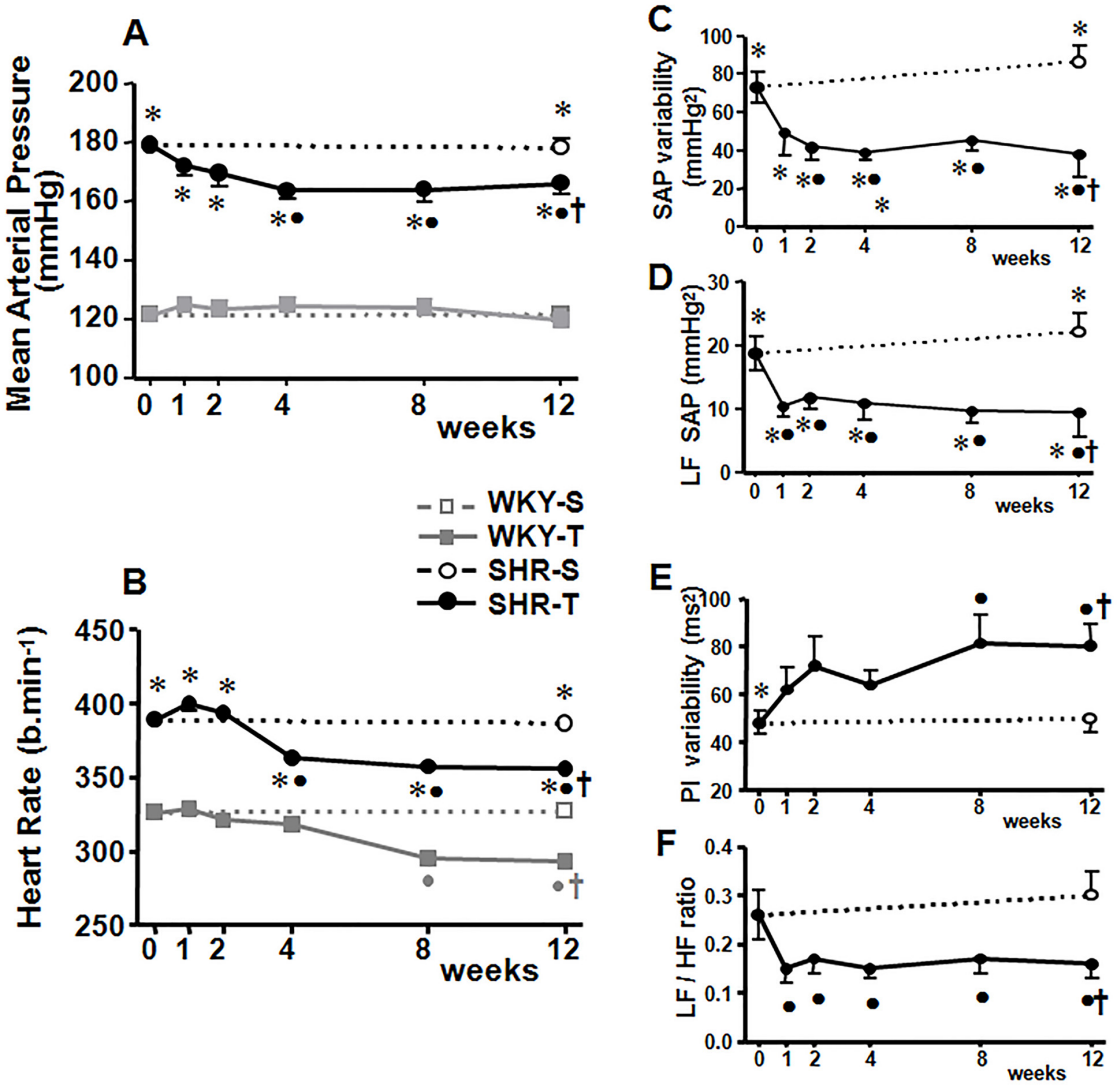
Training-induced changes on functional parameters. Sequential changes of resting mean arterial pressure and heart rate (panels **A** and **B** respectively) in trained (T) and sedentary (S) WKY and SHR (*n* = 9–16 rats/subgroup). Right panels depict time-course changes on systolic arterial pressure (SAP) variability (*panel* **C**), low frequency component of SAP (*panel* **D**), pulse interval (PI) variability (*panel* **E**) and low frequency to high frequency ratio of PI (*panel* **F**) of both groups during the 12-weeks protocols. Significances (P<0.05): * *vs* WKY, † *vs* S; ● *vs* week 0.

At the beginning of the experiments, SHR-S exhibited higher MAP and HR levels than age-matched WKY (week 0 in Fig 1A and 1B). Hypertension occurred simultaneously with increased systolic pressure variability, higher VLF, LF, and HF components and reduced PI variability (P<0.05 for SHR-S_0_ *vs*. WKY-S_0_, Table 2). Training caused significant reductions on resting HR in both groups, which appeared earlier in SHR (-6.6% at T_4_) than in WKY rats (-9.5% at T_8_, Fig 1B). In the SHR group there was a prompt and maintained trained-induced LF reduction accompanied by a slight HF increase, with a marked reduction in the LF/HF ratio to the heart that preceded the appearance of resting bradycardia and the significant increase in HR variability (Fig 1E and 1F). In the trained WKY, the establishment of resting bradycardia was preceded by transient, not maintained changes in the LF (reduction) and HF component (smaller age-induced reduction than in the WKY-S) of PI variability in such way that LF/HF ratio was significantly reduced (T_4_-T_8_) (data not shown). Progressive training-induced MAP fall was only observed in the SHR group, with a significant reduction from T_4_ to T_12_ (on average 8.1%, Fig 1A). This response was preceded by significant decreases in LF (T_1_, Fig 1D) and VLF (T_2_, data not shown) components of SAP as well as SAP variability (T_2_), whose reductions were maintained up to the end of protocols (Fig 1C and 1D, Table 2). Although no changes were observed in WKY rats kept sedentary, in the SHR-S there was a tendency for SAP variability, LF and VLF components of SAP and LF/HF ratio to the heart to increase during the 12-weeks protocol.

**Table 2:** Values of pressure variability (SAP var), heart rate variability (PI var) and respective spectral components at the beginning (week 0) and the end (week 12) of protocols in sedentary (S) and trained (T) WKY and SHR.

	Groups				
*Power spectral analysis*		WKY-S	WKY-T	SHR-S	SHR-T
**SAP var (mmHg** ^**2**^ **)**	w0	20.5±3.3		70.6±7.6*	
	w12	16.4±3.1	13.8±1.4	86.4±8.6*	39.3.8±7.7*●†
**HF–SAP (mmHg** ^**2**^ **)**	w0	1.5±0.3		5.81±1.2*	
	w12	1.2±0.2	1.1±0.1	6.2±0.8*	5.1±1.1*
**LF–SAP (mmHg** ^**2**^ **)**	w0	1.5±0.2		17.8±2.6*	
	w12	1.6±0.2	1.5±0.2	22.1±3.0*	11.0±2.6*†
**VLF–SAP (mmHg** ^**2**^ **)**	w0	4.8±0.7		27.1±6.5*	
	w12	5.9±0.9	5.7±0.7	35.5±6.1*	13.1±2.1*●†
**PI var (ms** ^**2**^ **)**	w0	70.9±7.6		48.0±4.9*	
	w12	69.4±9.1	63.2±6.3	50.0±6.1	80.6±9.6●†
**HF–PI (ms** ^**2**^ **)**	w0	19.8±2.4		13.1±1.6	
	w12	12.5±1.8	13.9±1.9	12.7±1.5	15.8±1.9
**LF–PI (ms** ^**2**^ **)**	w0	3.0±0.4		4.1±0.6	
	w12	3.0±0.5	2.1±0.3	4.9±0.9	1.6±0.2●†
**LF / HF ratio**	w0	0.18±0.03		0.26±0.05	
	w12	0.24±0.02	0.22±0.02	0.30±0.05	0.16±0.03●†

Values are means±SEM. Three-way factorial ANOVA. Significances (P<0.05): * *vs* WKY; † *vs* S; ● *vs* week 0.

### Time-course changes on Aogen, AT_1_ and Mas receptor in autonomic brain areas

**Fig 2:**
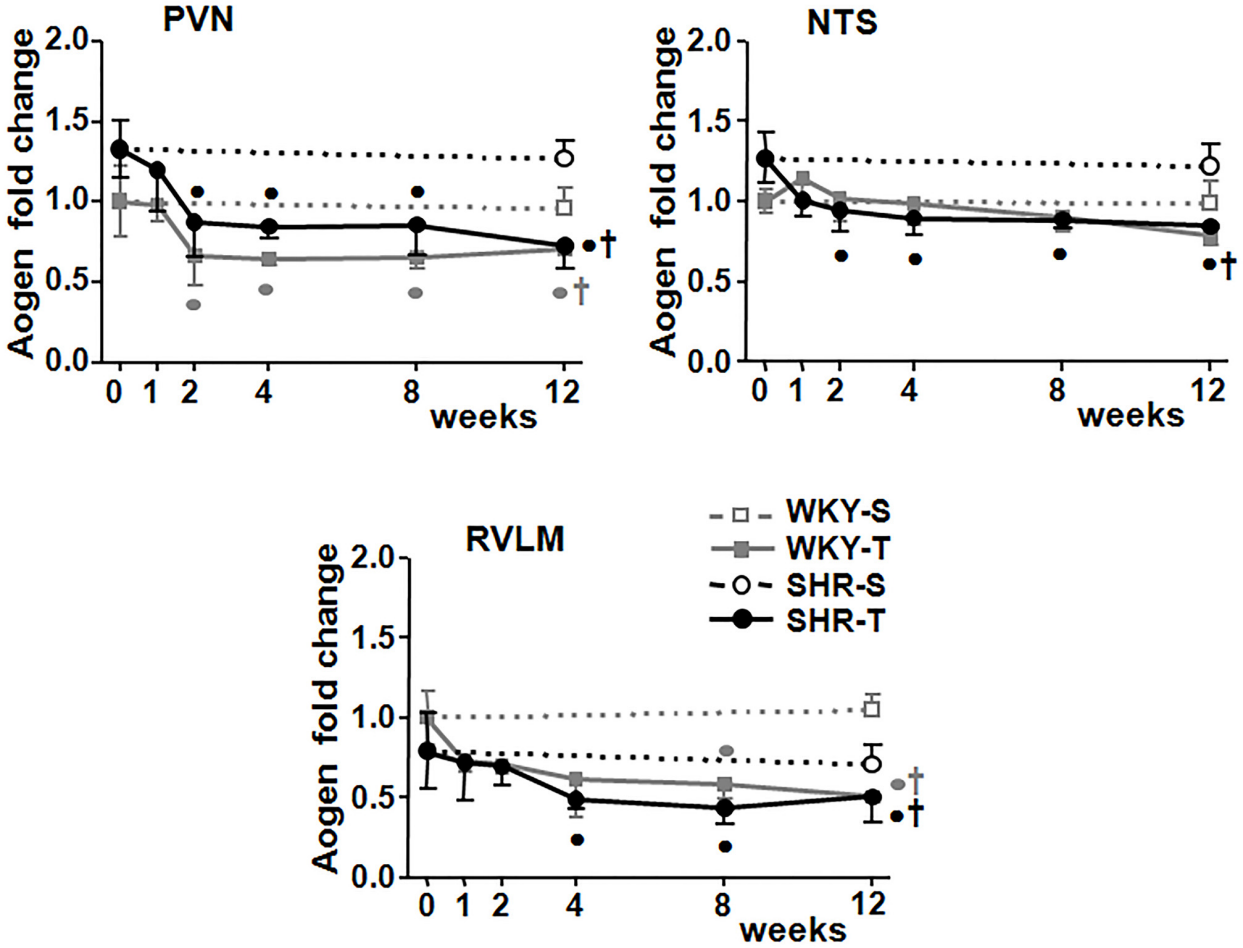
Training-induced changes on angiotensinogen (Aogen) expression in brain autonomic areas. Graphs show the sequential changes of Aogen mRNA expression within the PVN, NTS and RVLM of sedentary (S) and trained (T) WKY and SHR (*n* = 5–7 rats/subgroup). Significances (P<0.05): † *vs* S; ● *vs* week 0.

At the beginning of protocols SHR-S_0_ *vs*. WKY-S_0_ showed a tendency for increased Aogen mRNA expression in the PVN and NTS (+33% and +27%, P>0.05, respectively) with a slight, not significant, decrease in RVLM expression (-21%, Fig 2). In the SHR group, training was accompanied by a prompt reduction of Aogen mRNA expression in autonomic brain areas, starting at T_1_, but attaining significance at T_2_ in the PVN and NTS and at T_4_ in the RVLM (Fig 2). In all areas, reduced Aogen expression (on average -40%, -29% and -39% for PVN, NTS and RVLM, P<0.05, respectively) was maintained up to the end of training protocol. In the WKY group, training also determined significant reductions of Aogen mRNA expression in the PVN (from T_2_ to T_12_) and RVLM (T_8_-T_12_), without any change in the NTS (Fig 2). No changes were observed in the sedentary SHR and WKY groups during the 12-weeks protocol.

**Fig 3:**
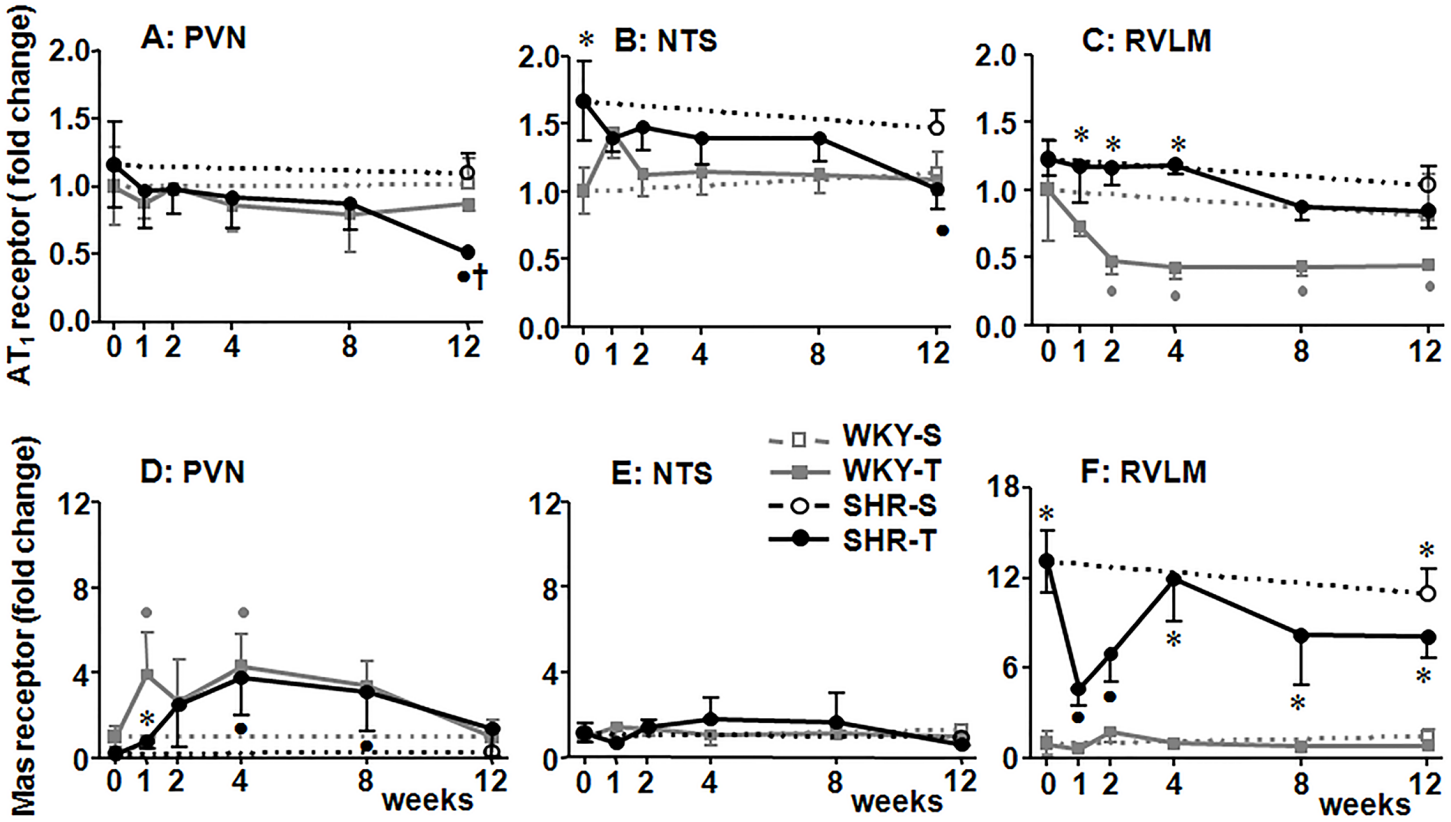
Training-induced changes on Angiotensin II type 1 receptor (AT_1_, *panels A*, *B and C*) and Mas receptor (*panels D*, *E and F*) mRNA expression in brain autonomic areas. Graphs show sequential changes within the PVN (panels **A** and **D**), NTS (panels **B** and **E**) and RVLM (panels **C** and **F**) of sedentary (S) and trained (T) WKY and SHR. (*n* = 5–8 rats/subgroup). Significances (P<0.05): * *vs* WKY, † *vs* S; ● *vs* week 0.

We also analyzed the sequential effects of training on mRNA expression of AT_1_ and Mas receptor in autonomic brain areas of SHR and WKY rats. SHR-S_0_ exhibited increased expression of AT_1_ in the NTS and of Mas receptor in the RVLM (P<0.05 when compared to respective WKY-S_0_, Fig 3B and 3F). In the SHR group training only caused a late reduction on AT_1_ mRNA expression in both PVN and NTS (significant after 12 weeks of training, Fig 3A and 3B), without significant changes in the RVLM. Trained SHR also exhibited transient, not maintained, changes in Mas receptor mRNA expression: a small increase in the PVN (T_4_-T_8_, Fig 3D) and a large reduction in the RVLM (T_1_-T_2_, Fig 3F), without changes in the NTS. In the trained WKY group, PVN Mas receptor was transiently increased (T_1_-T_4_, Fig 3D), while AT_1_ exhibited a large and maintained decrease in the RVLM (from T_2_ to T_12_, Fig 3C). Again, no changes were observed in the sedentary groups during the 12-weeks protocol.

**Fig 4:**
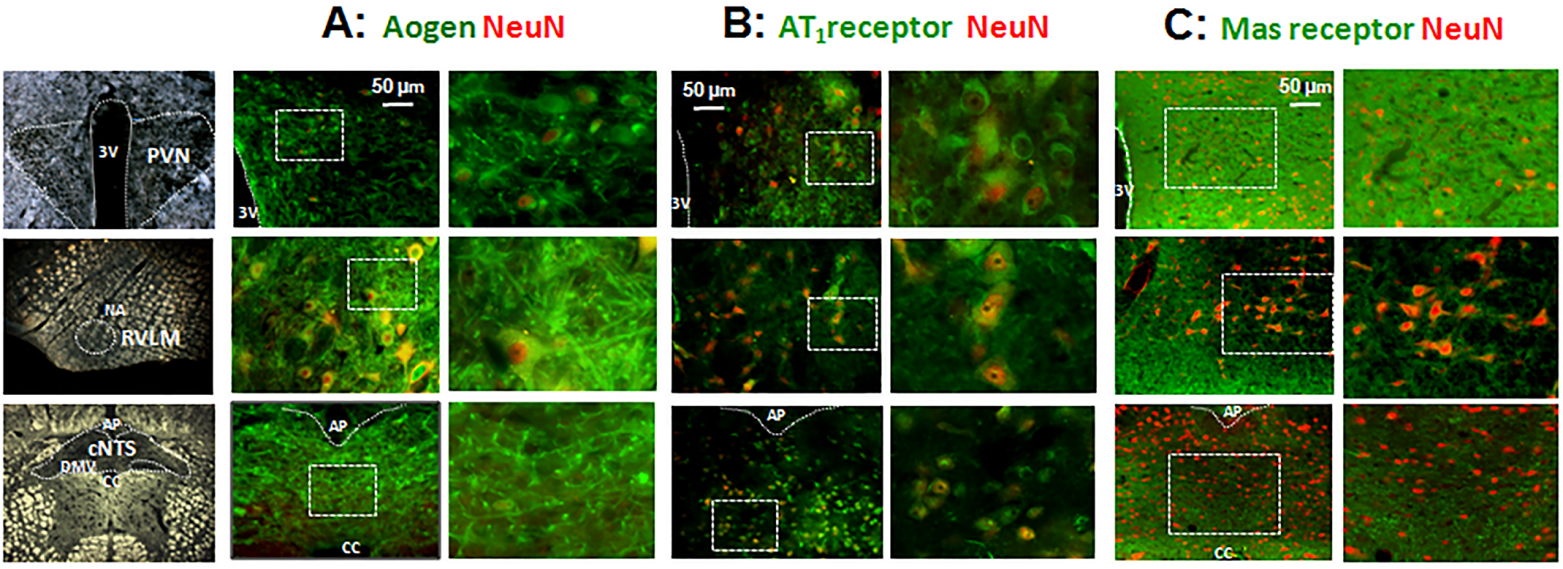
Photomicrographs illustrating the expression of angiotensinogen (Aogen, panel A), AT_1_ (panel B) and Mas Receptor (panel C) within the PVN (upper panels), RVLM (center panels) and NTS (lower panels) in sedentary SHR at the beginning of protocols. Double staining for the RAS precursor and receptors (green) and neuN (red). In each pair of pictures, the right represents an amplification of dashed rectangle on the left. In all areas observe neuronal and extra-neuronal expression of precursor and receptors.

**Fig 5:**
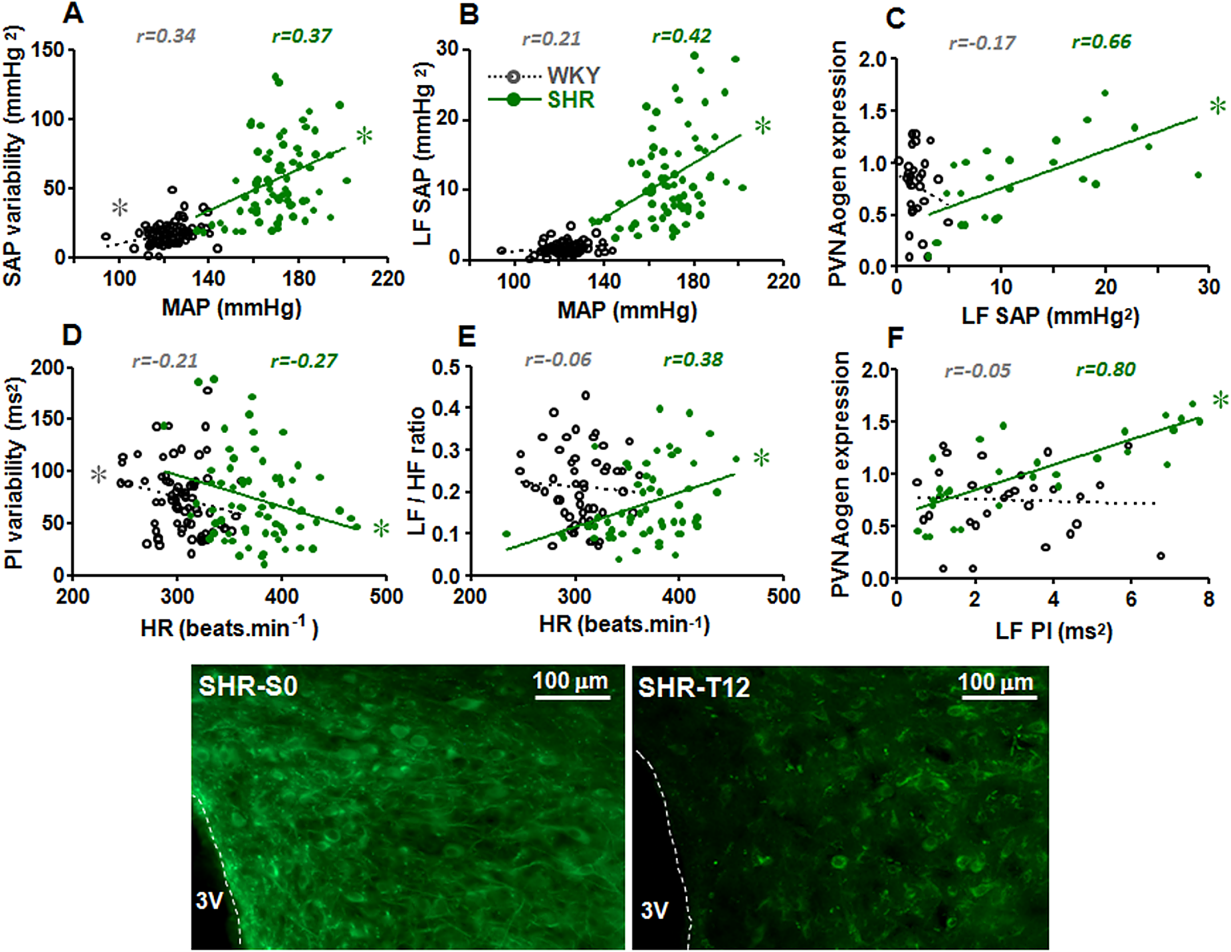
Correlations between functional parameters and PVN Aogen mRNA expression in WKY and SHR groups. *Upper panels* depict correlations between MAP and SAP variability (**A**), MAP and LF of SAP (**B**), LF of SAP and PVN Aogen expression (**C**), HR and PI variability (**D**), HR and LF/HF ratio (**E**) and LF of PI and PVN Aogen expression (**F**). Regression coefficients are shown in each panel and complete linear regression equations are presented in Table 3. * denotes a significant correlation (P<0.05). Photomicrographs (*lower panels)* compare the effect of training on PVN Aogen immunofluorescence in a sedentary SHR with a 12-weeks trained SHR. 3V, third ventricle.

To validate qPCR data, brains of some rats were processed for immunofluorescence assay. Sedentary SHR when compared to WKY-S (data not shown) exhibited dense Aogen content in neurons and glia within the medial PVN, RVLM and intermediate NTS (Fig 4A). In the SHR-S group, AT_1_ (Fig 4B) and Mas receptor (Fig 4C) were also expressed in neurons and glia within the PVN and NTS, with a dense expression in the glia. Interestingly, Aogen protein content was reduced in autonomic areas of SHR rats at the end of the protocols, with a marked reduction of PVN Aogen immunoreactivity in SHR-T_12_ when compared to SHR-S_0_ (Fig 5, lower panels).

### Correlations between functional parameters and the expression of brain RAS components

The comparison between functional changes and changes in the expression of RAS components induced by hypertension and training showed a great temporal coincidence between time-course changes of the cardiovascular responses and those of Aogen gene expression (early and marked reduction) in the three autonomic areas studied. As shown in Table 3 and Fig 5 (panels A, B and C), trained-induced MAP fall in the SHR group was positively correlated with the reduction of SAP variability and its LF component (indicative of decreased sympathetic vasomotor tonus) while SAP variability and LF decreases were positively correlated with trained-induced reduction on Aogen mRNA expression in the PVN of the trained SHR. In addition, HR reduction in the trained SHR was negatively correlated with increased PI variability and positively correlated with reduced sympatho-vagal balance (as indicated by LF/HF ratio) to the heart (Table 3, Fig 5D and 5E). The marked decrease in the variability of the sympathetic tonus to the heart (LF of PI, Fig 5F) and LF/HF ratio in the SHR were positively correlated with trained-induced reduction of Aogen expression in the PVN and RVLM and with AT_1_ mRNA expression in the RVLM (data on Table 3). In the WKY group, training-induced reduction of sympathetic component to the heart was positively correlated only with the reduced expression of AT_1_ in the RVLM (Table 3). No significant correlations were found between functional parameters and RAS components in the NTS and between cardiovascular responses and Mas receptor expression in the three autonomic areas analyzed (data not shown).

**Table 3:** Regression equations correlating training-induced changes on functional parameters with angiotensinogen and AT_1_ receptor expression in the brain autonomic areas of WKY and SHR groups.

Correlations	Groups	
	WKY	SHR
**MAP x SAP variability**	Y = 0.33x – 23*r = 0*.*341 P = 0*.*002*	Y = 0.75x – 71*r = 0*.*368 P < 0*.*001*
**MAP x LF SAP**	Y = 0.021x – 0.9*r = 0*.*211 P = 0*.*044*	Y = 0.200x – 21*r = 0*.*416 P < 0*.*001*
**SAP var x Aogen PVN**	Y = 0.004x + 0.69*r = 0*.*125 P = 0*.*565*	Y = 0.009x + 0.31*r = 0*.*657 P < 0*.*001*
**LF SAP x Aogen PVN**	Y = – 0.059x + 0.88*r = – 0*.*172 P = 0*.*381*	Y = 0.036x + 0.39*r = 0*.*655 P < 0*.*001*
**HR x PI variability**	Y = – 0.269x + 156*r = – 0*.*208 P = 0*.*043*	Y = – 0.304x + 188*r = – 0*.*271 P = 0*.*012*
**HR x LF/HF ratio**	Y = – 0.0002x + 0.28*r = – 0*.*061 P = 0*.*333*	Y = – 0.0008x – 0.13*r = – 0*.*376 P = 0*.*002*
**PI var x Aogen PVN**	Y = 0.001x + 0.64*r = 0*.*157 P = 0*.*417*	Y = 0.007x + 0.48*r = 0*.*630 P < 0*.*001*
**LF PI x Aogen PVN**	Y = – 0.011x + 0.79*r = – 0*.*053 P = 0*.*783*	Y = 0.121x + 0.60*r = 0*.*798 P < 0*.*001*
**LF PI x Aogen RVLM**	Y = – 0.036x + 0.64*r = – 0*.*127 P = 0*.*449*	Y = 0.118x + 0.10*r = 0*.*426 P = 0*.*024*
**LF PI x AT**_**1**_ **RVLM**	Y = 0.145x + 0.10*r = 0*.*618 P = 0*.*001*	Y = 0.054x + 0.92*r = 0*.*273 P = 0*.*176*
**LF/HF ratio x Aogen PVN**	Y = 0.717x + 0.60*r = 0*.*240 P = 0*.*218*	Y = 2.341x + 0.34*r = 0*.*761 P < 0*.*001*
**LF/HF ratio x AT**_**1**_ **PVN**	Y = 0.570x + 0.88*r = 0*.*114 P = 0*.*514*	Y = 1.450x + 0.56*r = 0*.*523 P = 0*.*006*

Aogen, angiotensinogen mRNA expression; AT_1_, angiotensin II type 1 receptor; HF, high frequency component; HR, resting heart rate; LF, low frequency component; MAP, resting mean arterial pressure; PI, pulse interval; PVN, paraventricular nucleus of hypothalamus; RVLM, rostroventrolateral medulla; SAP, systolic arterial pressure; var, variability. Correlations with functional data were made with 68–73 observations/group; correlations with mRNA expression data were made with 21–31 observations/group.

## Discussion

The present set of data confirmed the efficacy of exercise training to induce resting bradycardia in hypertensive and normotensive rats and to decrease partially blood pressure levels in the hypertensive strain. In addition, data revealed several new observations: 1) sedentary SHR when compared with age-matched WKY exhibited a clear tendency for higher Aogen mRNA expression in the PVN and NTS, augmented expression of AT_1_ in the NTS and a marked increase in Mas receptor expression in the RVLM; 2) two weeks of training are enough to reduce Aogen expression in the PVN and NTS of the SHR, while a significant decrease in the RVLM is only observed at the 4^th^ week; 3) training also causes a late AT_1_ decrease in the PVN and NTS of the SHR (observed only at T_12_) and induces transient not maintained changes in Mas receptor expression in the PVN (increased from T_4_-T_8_) and RVLM (reduced from T_1_-T_2_); 4) trained-induced Aogen reduction in the PVN of the SHR not only coincides, but correlates with both decreased pressure variability and reduced sympathetic vasomotor activity and with the reduction of sympathetic/parasympathetic ratio to the heart; 5) these changes in autonomic control of the heart and vessels precede both the installation of resting bradycardia and the significant blood pressure reduction in the hypertensive strain; 6) the decreased expression of RVLM AT_1_ (T_2_-T_12_) in trained WKY is correlated with the reduction of sympathetic activity to the heart and may contribute to the appearance of resting bradycardia in normotensive rats.

The majority of studies evaluating the effects of exercise on cardiovascular and autonomic parameters described blood pressure, heart rate and sympathetic/parasympathetic activity only at the end of the training protocol. Only few studies analyzed functional responses during the development of the training and sedentary protocols [19, 22]. Here, we analyzed time-course changes in trained and sedentary SHR and age-matched normotensive controls during a 12-weeks protocol. We showed that trained SHR exhibited a very rapid reduction in sympathetic activity to heart and vessels (1^st^ week, with reduction of sympathovagal balance to the heart) followed by reduced hormonal and pressure variability (2^nd^ week), which preceded the installation of resting bradycardia and pressure fall (occurring only after the 4^th^ week of training). In the WKY, training-induced autonomic adaptations were restricted to the heart, appeared later (around the 4^th^ week), but again preceded the appearance of resting bradycardia.

The efficacy of exercise training to reduce the overactivity of the ACE/Ang II-AT1 and to facilitate the effects of ACE2-Ang-(1–7)-Mas receptor axis has been demonstrated by several studies in hypertensive animals[20, 27, 28]. As for functional responses, these studies only analyzed the effects of training at the end of protocols and most of them linked training-induced changes in brain RAS expression/activity with the reduction of plasma catecholamine levels and/or blood pressure fall[5, 20, 29]. Here we recorded simultaneously the sequential cardiovascular responses, autonomic outflow to heart/vessels and training-induced changes in Aogen, AT_1_ and Mas receptor expression within the PVN, NTS and RVLM of hypertensive rats. There were different profiles, but the most consistent response observed was the decreased expression of Aogen mRNA in all autonomic areas, which exhibited different time-courses: it was promptly reduced in the PVN and NTS (significant changes from T_2_ on), showing a late decrease in the RVLM. Training was also accompanied by transient not maintained changes in Mas receptor expression (reduction from T_1_-T_2_ in the RVLM, increase from T_4_-T_8_ in the PVN) and by a slowly developing reduction of AT_1_ expression in the PVN and NTS that attained significance only after 12 weeks of training. Together these results indicated that the main effect of training in the SHR is a prompt down-regulation of brain RAS (by adjusting it to a lower level through the reduced availability of the precursor) while slowly reducing (PVN and NTS) or not changing (RVLM) the local density of AT_1_ receptors.

In the present study, we did not analyze the downstream effects of Ang II excess or of its blockade, but we recently reported that aerobic training corrected the autonomic dysfunction of the SHR by reversing the oxidative and pro-inflammatory profile within the PVN[19]. Taken together these observations suggest that training-induced reduction of RAS precursor (and therefore the decreased Ang II availability) is a main effect to blunt deficits in cardiovascular control exhibited by the sedentary SHR. Indeed in a recent report we demonstrated that exercise-induced down-regulation of vascular RAS in hypertensive rats was mainly caused by a rapid and marked decrease of the Aogen content in the renal artery[22]. Previous studies have already suggested a linkage between Aogen gene expression and human hypertension[30, 31]. In addition, availability of brain Aogen (mainly produced by the astrocytes and highly concentrated in the interstitial space and cerebrospinal fluid[4, 32]) has been linked to blood pressure levels since transgenic rats overexpressing Aogen gene exhibited high pressure levels[33] while transgenic rats with 90% decrease in astrocyte’s Aogen synthesis but unaltered plasma levels showed reduced blood pressure decrease[34]. It was also shown that pressure fall in hypertensive rats after chronic treatment with losartan[18] or following exercise training[5] was positively correlated with a robust reduction in Aogen expression in autonomic brain areas.

Although we hypothesized that training-induced functional benefits also depended on changes in RAS receptors’ expression, AT1 mRNA only correlated with LF of PI (within the RVLM of WKY) and with LF/HF ratio (within the PVN of the SHR). No correlation was observed between cardiovascular responses and Mas receptor expression. Indeed, AT_1_ was shown to be located pre-synaptically and post-synaptically in both glutamatergic and/or gabaergic interneurons[35–38], therefore mediating sympathoexcitation and/or sympathoinhibition, depending on the balance between these effects. In this regard Sheriff et al[39] described that blockade of AT_1_ within the RVLM was altered by the physiological condition: renal sympathetic nerve activity was unchanged in normoxic urethane anesthetized rats, increased during moderate hypoxia and decreased following local blockade with bicuculline. Shan et al [40] also demonstrated that within the NTS, AT_1_ inhibited further blood pressure elevation in the SHR, acting as a counterhypertensive mechanism involving inflammatory/angiogenic cells.

Our data also showed a compensatory increase of Mas receptor mRNA expression in the RVLM of the sedentary SHR and that changes in Mas receptor played a minor transient role in brain RAS adjustments induced by exercise training in this strain. Previous studies suggested that training-induced changes in ACE2-Ang-(1–7)-Mas receptor axis could be tissue-specific and differentially affected by the type, intensity and duration of the training protocol. It was shown that 16 weeks of treadmill training increased ACE2 and Mas receptor expression in the brain of the SHR[20], while 8 weeks did not change ACE2 expression and Ang-(1–7) content in both soleus and plantaris, but increased Mas receptor expression in the soleus and reduced it in the plantaris muscle[41]. It was also reported that swimming training increased the expression of ACE2 and Ang-(1–7) in the heart and augmented Mas receptor in the heart and aorta[42, 43]; on the other hand, a similar protocol was unable to induce changes in Mas receptor expression in the heart[44].

Confirming our previous observations[5, 18, 19, 25] low-intensity aerobic training causes a similar improvement on treadmill performance and resting bradycardia (a marker of training) in both groups, but downregulation of brain RAS-related improvements on cardiovascular system were only observed in the hypertensive strain. Therefore, regular physical activity is an useful tool for the treatment of hypertensive as well as other patients with cardiovascular disease[21].

Taken together the present set of data indicated that exercise-induced down-regulation of brain RAS in hypertensive rats is a prompt response to training caused by a marked reduction on the availability of the precursor mainly in the PVN, with minor changes in the activity of RAS counter-regulatory axes. Indeed training-induced reduction on PVN Aogen expression in SHR occurred simultaneously and was positively correlated with the reduced sympathetic outflow to the periphery, while the smaller sympathetic activities to heart and vessels were significantly correlated with reductions in SAP and PI variabilities, resting HR and MAP fall. In this regard, recent studies in SHR showed that training reduced brain Ang II availability, decreased AT_1_ activation and caused a marked reduction in both PVN oxidative stress and pro-inflammatory profile, thus improving cardiovascular control[19, 20]. From these results we suggest that down-regulation of RAS system by exercise training in hypertensive individuals occurs in several brain autonomic areas, but the reduced PVN Aogen availability seems to be the main effect to blunt hypertension-induced deficits in cardiovascular control driven by vasoconstrictor, trophic, pro-oxidative and pro-inflammatory effects of Ang II excess. In normotensive rats training also decreases brain RAS activity by reducing AT_1_ expression in the RVLM, which correlates positively with the decrease in sympathetic activity and the appearance of resting bradycardia.
